# Sport and academic engagement of 1,387 Slovenian dual-career athletes before and during COVID-19 lockdown—what did we learn?

**DOI:** 10.3389/fpsyg.2023.1173261

**Published:** 2023-05-12

**Authors:** Kristina Drole, Armin Paravlic, Jay Coakley, Mojca Doupona

**Affiliations:** ^1^Faculty of Sport, University of Ljubljana, Ljubljana, Slovenia; ^2^Sociology Department, University of Colorado, Colorado Springs, CO, United States

**Keywords:** COVID-19 pandemic, education, sport policy, student-athlete, training load, academic load, behavioral changes

## Abstract

**Introduction:**

Since the coronavirus disease outbreak in 2019, there have been several preventive measures and restrictions applied to minimize the transmission of the virus. While lockdown has affected our everyday lives, it has negatively impacted sport and athletes as well.

**Methods:**

1,387 Slovenian dual-career (DC) athletes (47.4% females, 52.6% males) participated in the 22-item questionnaire to gather information on their sports and academic engagement before and during COVID-19 lockdown period. Half of the athletes were enrolled in education at the secondary level (*n* = 819, aged 15–18  years), while the others were enrolled in primary (*n* = 301, 8–14 years) and tertiary (*n* = 267, 19–36  years) education. All participants in the current study have a valid athlete categorization by the Slovenian Olympic Committee and are competing at either junior (31.7%), national (26.9%), prospective (29.5%), international (8.5%), world (2.3%) or Olympic (1.2%) level.

**Results:**

DC athletes spent less time on training (−4.7 h; *p* < 0.001), learning (−1.0 h; *p* < 0.001), exams (−0.9 h; *p* < 0.001), laboratory work (−0.6 h; *p* < 0.001), and other educational activities (−0.3 h; *p* < 0.001) during COVID-19 lockdown compared to period before the lockdown. Their training environment was changed so they trained either at home or outdoors. Results showed that indoor (−3.7 h; *p* < 0.001) and team sport athletes (−1.3 h; *p* < 0.001) trained less than outdoor and individual sports. Male athletes spent more time on training both before (1.3 h; *p* < 0.001) and during lockdown (1.3 h; *p* < 0.001) and other sport-related activities (1.3 h; *p* < 0.001). On the other hand, female athletes spent more time on studying both before (1.5 h; *p* < 0.001) and during lockdown (2.6 h; *p* < 0.001). Both sport and educational activities were influenced by athletes’ age (*p* ≤ 0.017).

**Conclusion:**

Indoor and team sport athletes were more affected by the governmental measures than outdoor and individual sport athletes. Male athletes experienced a greater decline in learning time compared to female athletes. DC is shown to be beneficial for athletes even in times of COVID-19 lockdown, as DC athletes report smaller decline in motivation, shifting attention from sport to study and having fewer mental problems due to uncertain sports future. The feedback of the preventive measures could serve to assist policy makers and athlete’s support staff to form and apply preventive measures that are more effective for DC athletes’ training and education.

## Introduction

Beginning in 2020, we faced a new virus without knowledge of its severity and consequences. In response, several preventive measures were applied by the government. Coronavirus disease (COVID-19) greatly influenced all aspects our lives during 2020 and 2021 (Fedyk et al., 2022). During the first few days of the disease occurrence in the beginning of March, the epidemiological situation did not dictate emergency measures, therefore there were no special restrictions of the daily activities both in the work environment and in everyday life. At that time, the only recommended preventive measures included washing hands, sanitizing, not touching eyes and mouth, and restricting contacts with people showing any symptoms of a respiratory disease. Yet, the COVID-19 disease spread faster than anyone anticipated, which caused most countries, including Slovenia, to apply a strict lockdown by closing educational institutions and non-essential industry, and restricting sports-training (Zhang et al., 2020).

In the light of COVID-19 lockdown, schools and universities closed their doors, resulting in students spending more time at home and meeting their study obligations there. This changed family daily routines and sleeping habits (López-Bueno et al., 2020; Moore et al., 2020; Fasano et al., 2021; Segre et al., 2021). The earliest works investigating the consequences of COVID-19 induced lockdown on physical and mental wellbeing showed changes in people’s emotional state. These included feelings of worry, fear and longing due to lack of social contact (Fasano et al., 2021), along with increased screen time and lack of physical activity (LeBlanc et al., 2015; Ammar et al., 2020; Clemente-Suárez et al., 2020; López-Bueno et al., 2020; Moore et al., 2020). These negative consequences were also observed in athletes (Mehrsafar et al., 2021; Paravlic et al., 2022). However, a special subgroup of athletes are dual-career (DC) athletes who coordinate regular study obligations with their sports-related obligations. Even before the COVID-19 pandemic occurred, balancing the academic and sports career was a challenging task for DC athletes. Their training and competition schedule usually includes 20–30 h of training per week (Aquilina, 2013), along with frequent international travel (Kerštajn and Topič, 2017) and the same amount of study-related obligations.

Article 35 of the Sports Act (2017) stipulates that athletes are entitled to the adjustment of school and study obligations, and their scope and manner of adjustment are determined by regulations governing the field of education. In the field of secondary education, DC athletes have the opportunity to adapt their education to more effectively coordinate their sports and school obligations. However, the adjustments for DC athletes are still not well resolved at the university level, where the Higher Education Act stipulates that higher education institutions determine the study regime, forms and periods of knowledge testing by themselves. Therefore, the adjustments for DC athletes at tertiary (university) level of education are still very heterogeneous, so that students from different study programs at the same level of competition still lack the same conditions for studying. However, DC has several positive benefits and is strongly recommended for athletes, as it may add to both future career opportunities and easier transition to the labor market (Barriopedro et al., 2018). A study on 15 former Olympic athletes found that athletes with dual-career adjusted better and had less difficulty reintegrating into society after sport’s career termination than athletes who solely prioritized sport (Torregrosa et al., 2015). Athletes from two previous studies (Price et al., 2010; Torregrosa et al., 2015) claim that DC helped them to achieve well-being and a well-rounded life during their athletic career. Furthermore, changing from a mostly mental activity (study) to a physical one (sport) can act as a form of recovery from the other activity, and benefits athletes in the course of their dual-careers (Stambulova et al., 2015).

Due to a possibility of increased risk of disease transmission associated with high-intensity physical activity, national governments and international sporting committees implemented COVID-19 measures, canceling sport participation and events (Parnell et al., 2022). This caused several alterations in the training regimes, such as training frequency, duration, intensity and training environment change. As a result, changes in training and study regimes impacted the physical and mental well-being of athletes (Mehrsafar et al., 2021; Paravlic et al., 2022). However, DC athletes might respond differently to the pandemic due to the added stressors of their dual careers. Therefore, variations in these study and training alterations by DC athletes with different socio-demographic and sports-related characteristics remain unknown and should be investigated.

As COVID and other virus outbreaks remain ongoing, a detailed analysis of the current well-being of athletes is needed to provide policy makers and athletes’ support staff with information about the impact of preventive measures. Understanding how they have adapted their training and competition plans, and the impact of these, can help inform strategies for future disruptions. Therefore, the aim of this study was to (i) investigate the effects of COVID-19 lockdown on DC athletes’ training and education, (ii) identify whether the sports-related and socio-demographic characteristics of the athletes influenced differences in their training and educational activities at various times during the pandemic, and (iii) investigate the benefits of dual-career for coping with the COVID-19 pandemic.

## Materials and methods

Currently, there is no available questionnaire used to collect relevant data on how DC athletes coped during the COVID-19 lockdown period and how they adapted their daily activities to maintain their athletic and educational competitiveness. Based on previous studies available (Izzicupo et al., 2021), a questionnaire was developed.

### Procedures

This longitudinal observational cohort study was conducted to investigate differences in sports and academic engagement between the time before (PRE_LD_) and during COVID-19 lockdown (DUR_LD_) (starting on 13.3.2020) in DC athletes. To identify variations among the athletes, a group of researchers developed a questionnaire customized for a Slovenian speaking population. For the primary purpose of the study, the initial administration of the questionnaire occurred during the first week of September 2020 (Time 1). However, to investigate the reliability of the questionnaire, it was administered again one week later (Time 2). At both time points, the questionnaires were administered through the online platform 1 ka.^1^

### Study sample

To be included in the current study, participants were required to fulfil the following inclusion criteria: (a) to be an active athlete, categorized by the Slovenian Olympic Committee (SOC) regulations; (b) to be simultaneously involved in some form of organized education. Athletes were included regardless of age, sex, education level, type of sport, and competition environment. SOC categorization is based on the results achieved by an athlete and his/her level of competition. Thus, athletes are categorized to either junior, national, prospective, international, world or Olympic level. Currently, there are 7,780 athletes in Slovenia with valid SOC categorization (Slovenian Olympic Committee, 2023). All participants were informed about the aims of the study and were asked to provide a written consent. This study was approved by the Ethics Committee of the Faculty of Sport (University of Ljubljana), number: 033-52/2022-4, and all procedures were carried out in agreement with the Declaration of Helsinki.

### The instrument—questionnaire

The questionnaire was designed to gather information from the athletes about their sports and academic engagement before and during COVID-19 lockdown period.

The questionnaire was constructed by a group of researchers (KD, AP, and MD) to elicit answers for the current research question. In the first phase, each researcher prepared several potential questions. In the second phase, each question was thoroughly reviewed and checked for understanding by all group members. The third phase consisted of a pilot study, in which 15 randomly selected subjects (researchers, students and athletes) completed a test version of the questionnaire. After developing consensus on the final version of the questionnaire, MD developed an online version of the questionnaire and sent it to the participants.

The questionnaire consists of 22 questions in each of the following categories:

Sociodemographic (Q1–Q5);

Sport and academic engagement before and during COVID-19 lockdown period (Q6–Q14);

Support and benefits of dual-career (Q15–Q22).

The sociodemographic characteristics section includes the following questions: sex, age, current level of education, sport and level of sport competition by the SOC ranking. The sport and academic engagement section consisted of nine questions related to hours of sport and educational activities before and during COVID-19 lockdown. They were asked if they trained/went to school, how was their training/education changed and the reasons for not participating in the training process during the lockdown. The support and benefits of dual-career section includes questions about DC athletes’ perceptions and response to the pandemic, including why they think DC helped/did not help them to cope with the pandemic. The questions were mostly closed-ended, while in some cases the athletes could write their own answer if it wasn’t provided among the choices.

### Statistical analysis

All data were presented as means (±SD) with 95% confidence intervals and mean difference (MD) where applicable. The statistical analyses were conducted using SPSS statistical software (version 27.0, IBM Inc., Chicago, United States). Normality of data distribution was confirmed by the Shapiro–Wilk test, while the homogeneity of variances was tested using the Levene’s test for all dependent variables. To investigate the reliability of the questionnaire, the relative reliability of all dependent variables between Time 1 and Time 2 was estimated using the intra-class correlation coefficient (ICC), two-way random model (consistency type). ICC values were considered as very high if >0.90, high if between 0.70 and 0.89, and moderate if between 0.50 and 0.69. Additionally, a standard error of estimate (SEM) followed by the coefficient of variation (CV) were calculated as measures of absolute reliability, which indicates within subject variation, as previously suggested (Hopkins, 2000). Results showed that average ICC values ranged from 0.787 (Q13) to 1.000 (Q2).

To answer our primary objective and assess the influence of independent variables such as type of sport (indoor vs. outdoor), categorization (junior vs. national vs. prospective vs. international vs. world class vs. Olympic), sex (males vs. females) and age (8 to 14 years of age vs. 15 to 18 years of age vs. 19 to 36 years of age) on DC athletes’ engagement in sports and educational activities at PRE_LD_ and DUR_LD_, a Kruskal–Wallis test followed by Mann–Whitney test was applied. Additionally, to compare differences in observed changes (∆) in sports and academic engagement between PRE_LD_ and DUR_LD_, a Mann–Whitney test was applied. For the post-hoc analysis, a Bonferroni adjustment for value of p interpretation was used. For all analysis conducted, the statistical significance was accepted at *p* < 0.05.

## Results

### Athletes’ socio-demographic characteristics

Athletes’ socio-demographic characteristics are presented in Table 1. A total of 1,387 Slovenian DC athletes (47.4% females, 52.6% males; 8–36 years of age; mean age = 17 ± 5.14 years) participated in the study. Approximately half of the athletes are enrolled in education at the secondary level (*n* = 819, 15–18 years of age), while the other half is participating in primary (*n* = 301, 6–14 years of age), and tertiary (*n* = 267, from 19 years of age on) education. All athletes in the current study have a valid categorization by the SOC which is the basis for obtaining the status rights of athletes, and are therefore competing at either junior (31.7%), national (26.9%), prospective (29.5%), international (8.5%), world (2.3%) or Olympic (1.2%) level. For the purpose of this study, we split the cohort of athletes in subcategories according to type of sport (individual = 69.3%, or team = 30.7% sport), competition environment (outdoor = 34.6% or indoor = 65.4% sport) and three age groups: first age group (8–14 years), second age group (15–18 years) and third age group (19–36 years).

**Table 1:** Sociodemographic characteristics of participants (*N* = 1,387).

Characteristics		*N*	Percent (%)
Sex			
	Female	657	47.4
	Male	730	52.6
Current level of education			
	Not in the education process	0	0.0
	Primary school	301	21.7
	High school	698	50.3
	Vocational school	121	8.7
	Bachelor’s study	216	15.6
	Master’s study	47	3.4
	Doctoral study	4	0.3
Ranking by the SOC			
	Junior	402	31.7
	National	341	26.9
	Prospective	375	29.5
	International	108	8.5
	World	29	2.3
	Olympic	15	1.2
Type of sport			
	Individual	961	69.3
	Team	426	30.7
Competition environment			
	Indoor	906	65.4
	Outdoor	480	34.6

#### DC athletes’ engagement in sports and educational activities before and during the COVID-19 lockdown

**Figure 1:**
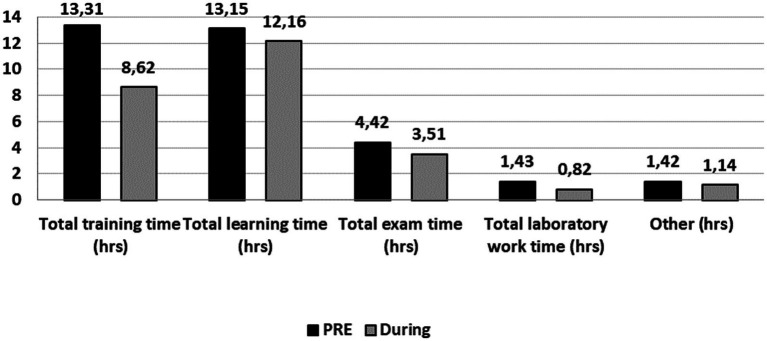
Comparisons of sports and educational activities before (PRE_LD_) and during (DUR_LD_) lockdown.

When compared to PRE_LD_, DC athletes spent less total time on training (−4.7 h; *Z* = −23.464; *p* < 0.001), learning (−1.0 h; *Z* = −4.806; *p* < 0.001), exams (−0.9 h; *Z* = −11.737; *p* < 0.001), laboratory work (−0.6 h; *Z* = −11.742; *p* < 0.001), and other educational activities (−0.3 h; *Z* = −5.888; *p* < 0.001) (Figure 1) DUR_LD_.

#### Differences in DC athletes’ engagement in sports and educational activities before and during the COVID-19 lockdown considering athletes’ age

**Figure 2:**
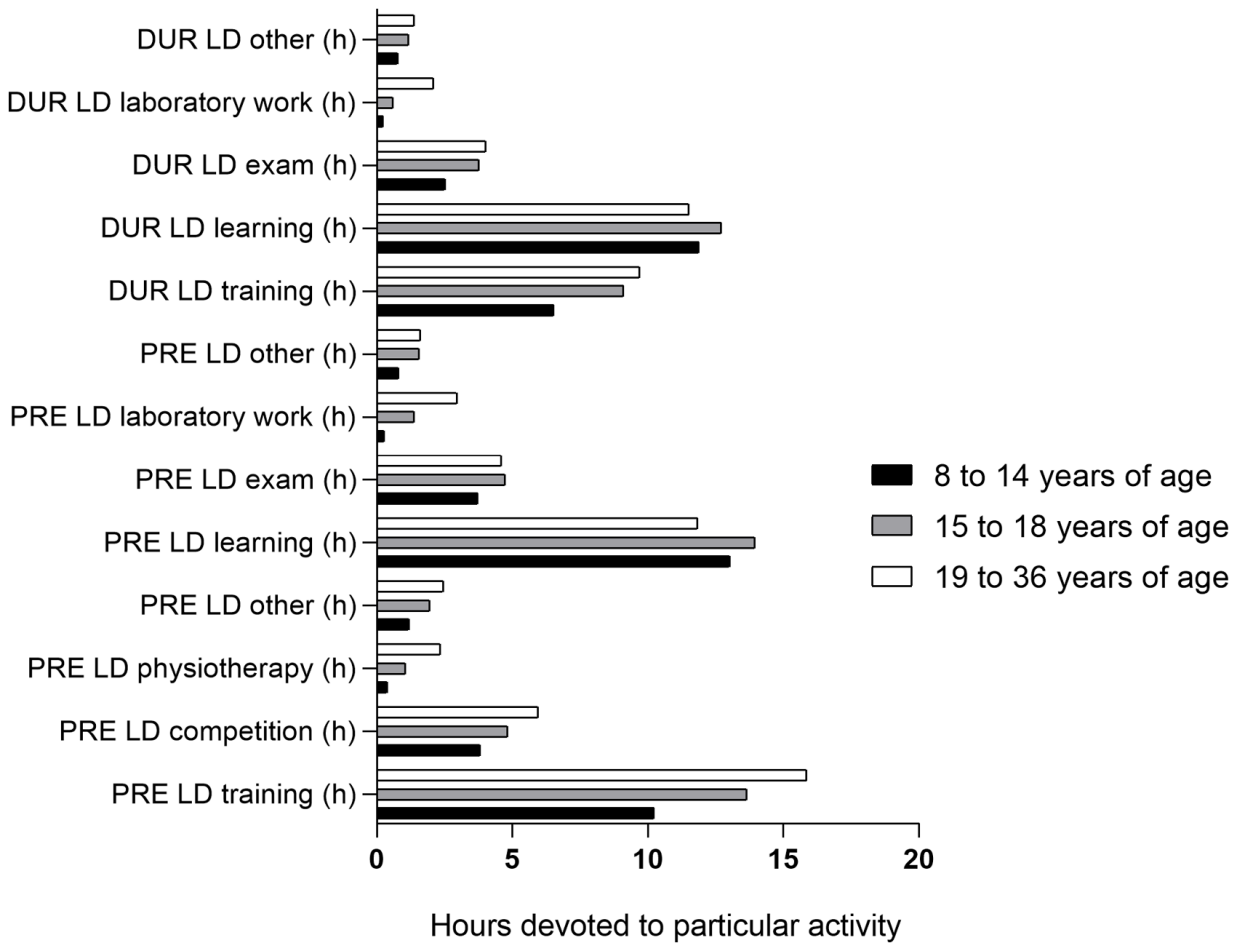
Comparisons of sports and educational activities before (PRE_LD_) and during (DUR_LD_) lockdown according to age.

Both sport and educational activities were influenced by athletes’ age [H ranging from 8.119 (learning hours, *p* = 0.017) to 114.345 (training hours, *p* < 0.001)] (Figure 2). The results showed that athletes in the first age group (8–14 years) spent significantly less time on all activities before and during lockdown, compared to other age groups (*p* < 0.001 for all analyes), except in learning time both before and during lockdown as well as other educational activities that did not differ compared to the other two groups. When differences between two older groups were considered, a third age group (19–36 years) spent more time in training, competing, physiotherapy, other physical activities, learning, laboratory work and other educational activities, compared to the second age group (15–18 years) (Figure 2).

**Figure 3:**
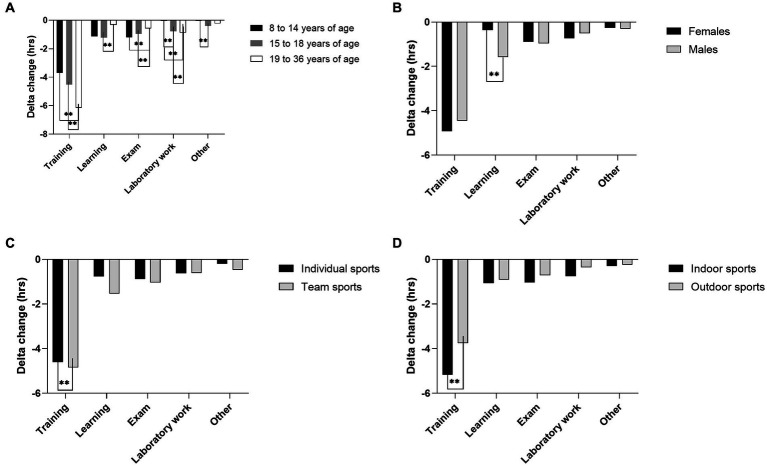
Before (PRE_LD_) to during (DUR_LD_) lockdown changes in athletes’ engagement in sport and academic activities according to **(A)** age, **(B)** sex, **(C)** type of sport and **(D)** competition environment.

Moreover, when difference in time spent on different activities from PRE_LD_ to DUR_LD_ were considered, results reached a significance for training time (*H* = 20.739, *p* < 0.001), time used on exams (*H* = 5.981, *p* = 0.016), laboratory work (*H* = 52.975, *p* < 0.001) and other educational activities (*H* = 12.977, *p* = 0.002). In detail, the second age group (15–18 years) experienced greater reduction in laboratory work hours (percent difference [PD] = 181.5%; *Z* = −7.502; *p* < 0.001) and hours of other educational activities (PD = 166.3%; *Z* = −3.728; *p* < 0.001), compared to the first age group. Also, when compared to the third age group, the first age group experienced greater reduction in time spent on tests (PD = 70.1%; *Z* = −2.838; *p* = 0.005). In contrary, they experienced lower decrease in training hours (PD = 50%; *Z* = −4.480; *p* < 0.001) and laboratory work (PD = 183.7%; *Z* = −3.561; *p* < 0.001) from PRE_LD_ to DUR_LD_ when compared to the third age group. Finally, compared to second age group, third age group showed a greater reduction in time spent in training (PD = −30.5%; *Z* = −3.212; *p* = 0.001) and laboratory work (PD = −13.1%; *Z* = −2.457; *p* = 0.014) from PRE_LD_ to DUR_LD_. In contrary, they showed a lower decrease in time spent in learning (PD = 117.2%; *Z* = −2.431; *p* = 0.015) and tests (PD = 47.6%; *Z* = −2.517; *p* = 0.012) compared to the second age group, from PRE_LD_ to DUR_LD_ (Figure 3A).

#### Differences in DC athletes’ engagement in sports and educational activities before and during the COVID-19 lockdown considering athletes’ sex

**Figure 4:**
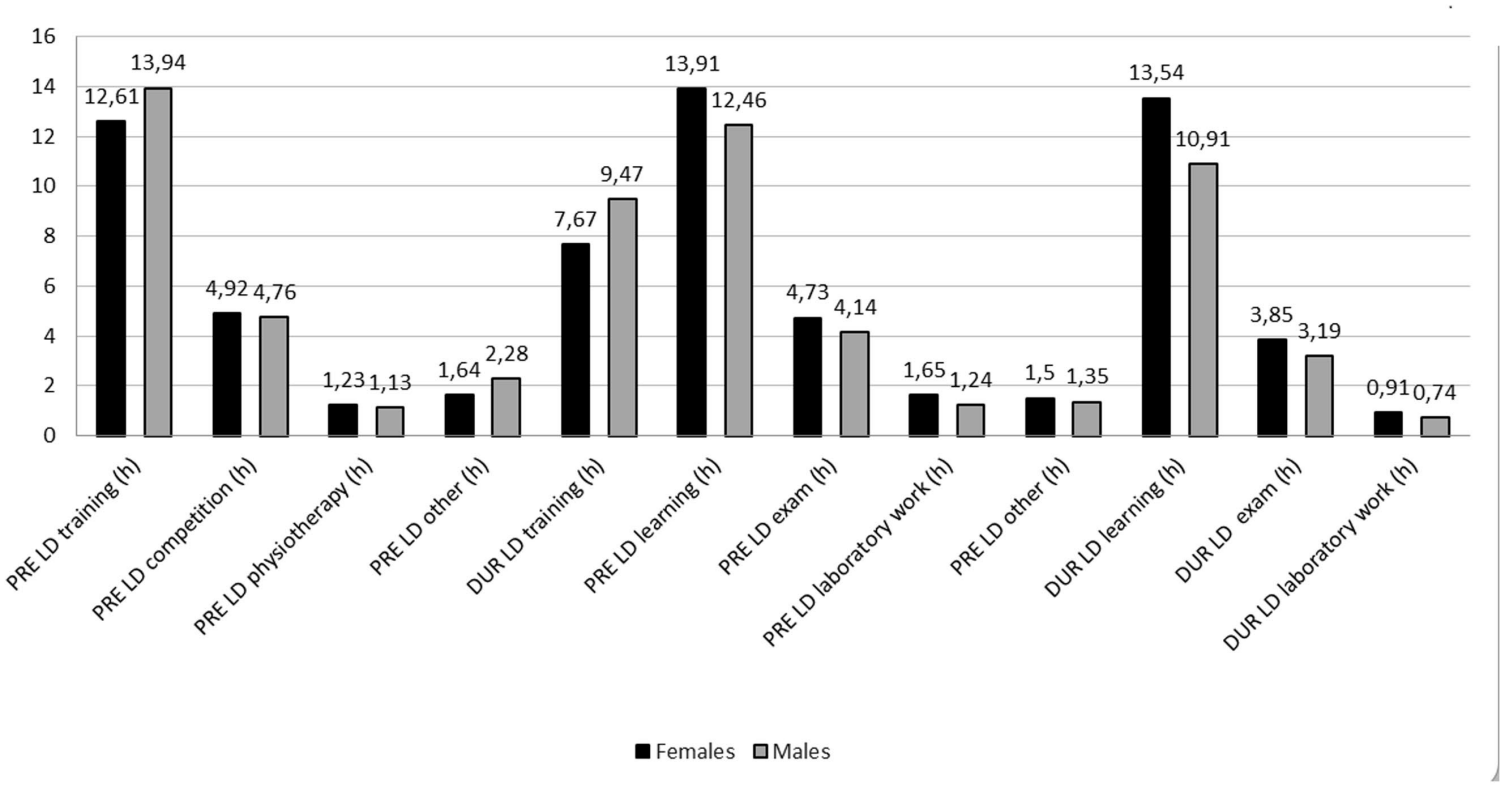
Comparisons of sports and educational activities before (PRE_LD_) and during (DUR_LD_) lockdown between sex.

Both sport and educational activities were influenced by athletes’ sex. The results showed that compared to females, male athletes spent more time on training before lockdown (1.3 h; *Z* = 11.742; *p* < 0.001), other sport-related activities (1.3 h; *Z* = 11.742; *p* < 0.001) and training during lockdown (1.3 h; *Z* = 11.742; *p* < 0.001). On the other hand, male athletes spent less time on studying both before (−1.5 h; *Z* = −3.602; *p* < 0.001) and during lockdown (−2.6 h; *Z* = −5.358; *p* < 0.001), total laboratory work before lockdown (−0.4 h; *Z* = −2.049; *p* = 0.040), and exams before (−0.6 h; *Z* = −3.558; *p* < 0.001) and during lockdown (−0.7 h; *Z* = −2.627; *p* = 0.009) (Figure 4). Compared to female athletes, male athletes experienced significantly greater decline in learning activities [percent difference (PD) = 123.9%; *Z* = −2.785; *p* = 0.005] from PRE_LD_ to DUR_LD_ (males ∆ = −1.58 ± 7.79 h. vs. females ∆ = −0.37 ± 7.80 h) (Figure 3B).

#### Differences in DC athletes’ engagement in sports and educational activities before and during the COVID-19 lockdown considering level of competition

**Figure 5:**
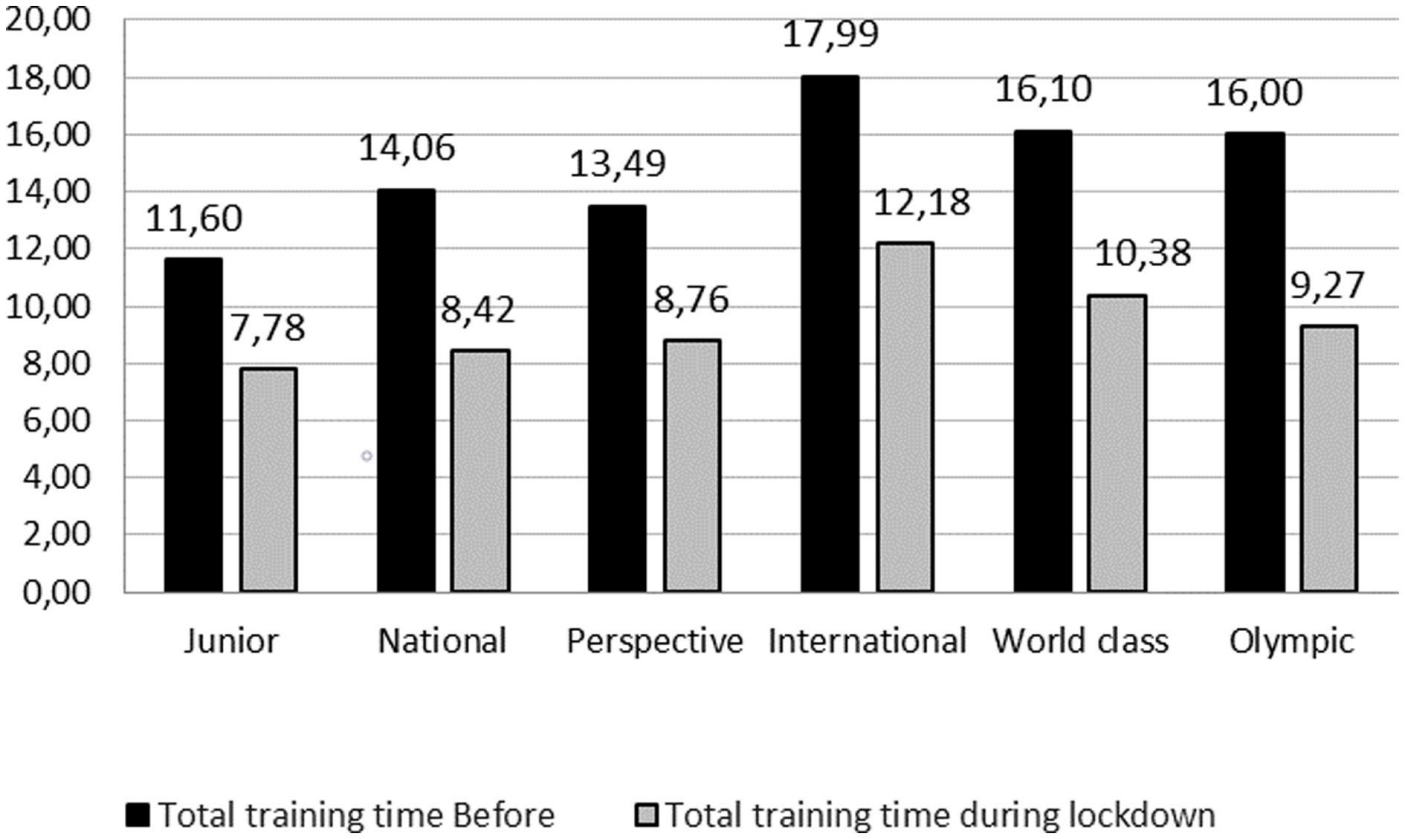
Comparisons of average weekly training time before (PRE_LD_) and during (DUR_LD_) lockdown between athletes competing at different levels.

**Figure 6:**
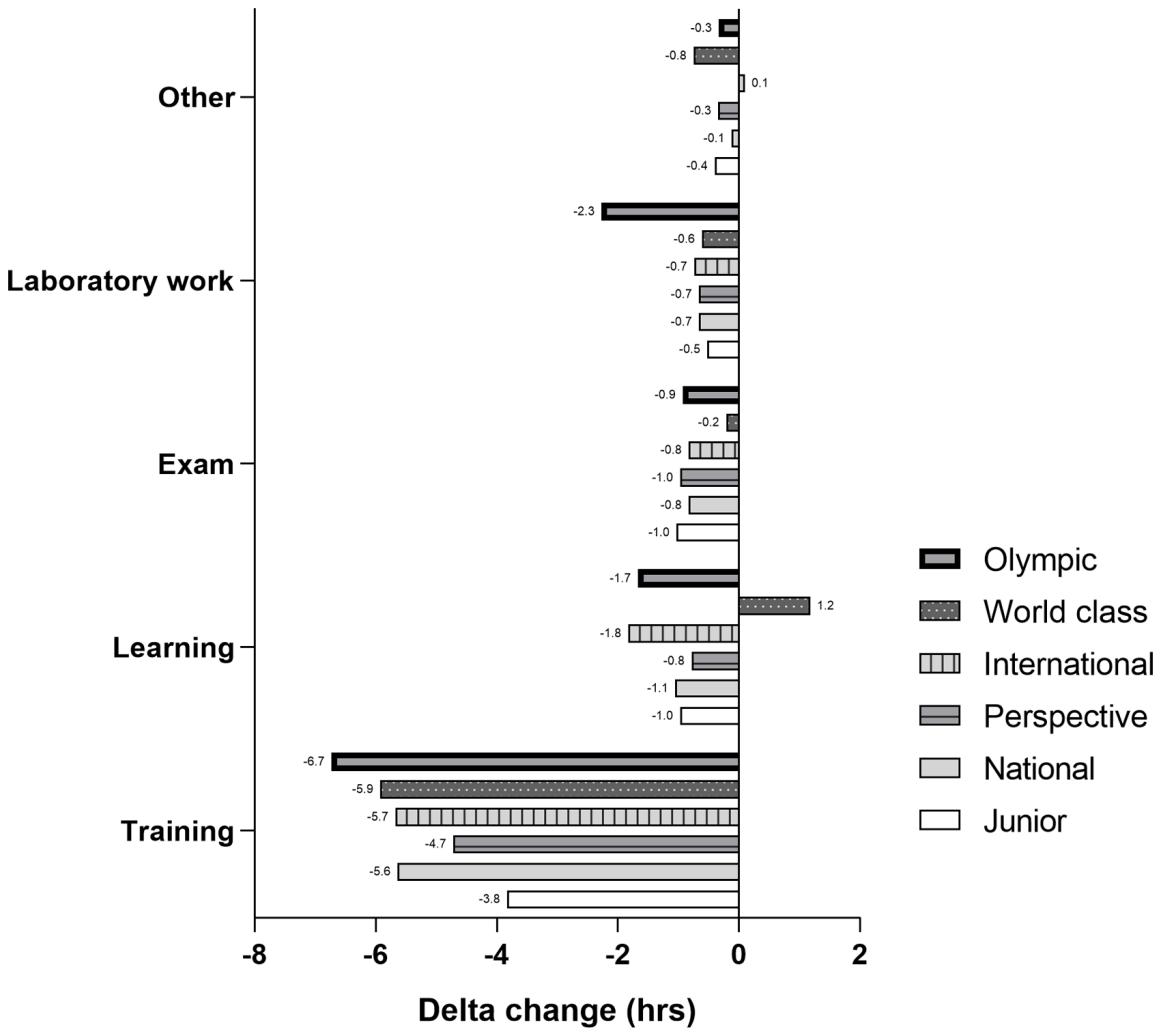
Comparisons of sports and educational activities before (PRE_LD_) and during (DUR_LD_) lockdown according to level of competition.

Total training time both before and during a lockdown differed between athletes who participated in different levels of competition (*H* = 83.157; *p* < 0.001) (Figure 5). Thus, results showed that junior athletes spent less time on training before lockdown (−2.5 h; *Z* = −4.920; *p* < 0.001) compared to National level athletes. Also, when compared to prospective athletes, junior athletes spent less time on training both before (−1.9 h; *Z* = −4.257; *p* < 0.001), and during lockdown (−1.0 h; *Z* = −2.512; *p* = 0.012). As it is seen from the figure, all the athletes trained less DUR_LD_ compared to PRE_LD_ regardless of their competition level. Furthermore, when differences in time spent on different activities from PRE_LD_ to DUR_LD_ were considered, results reached a significance for training time only (*H* = 21.229, *p* = 0.001). Post-hoc analysis showed that junior athletes experienced less decline in training time from PRE_LD_ to DUR_LD_ compared to national level athletes (PD = −38.2%; *p* < 0.001), but not other groups of athletes. There were no significant differences between other groups (Figure 6).

#### Differences in DC athletes’ engagement in sport and education-related activities before and during the COVID-19 lockdown considering type of sport

**Figure 7:**
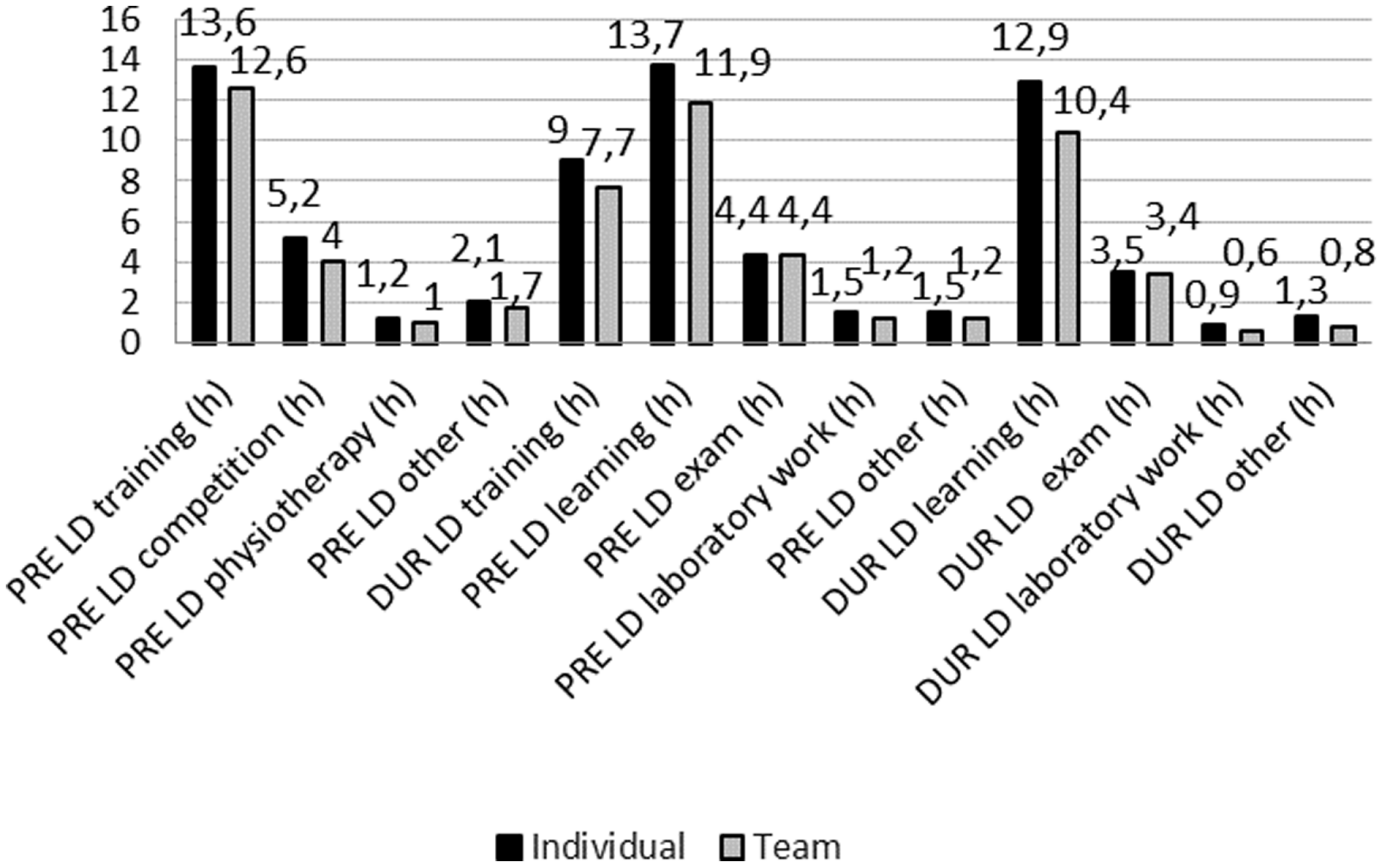
Comparisons of time spent on sport and educational activities before (PRE_LD_) and during (DUR_LD_) lockdown between athletes competing in individual and team sports.

When looking at individual and team sport athletes, the results showed that athletes in individual sports spent more time on sport and educational activities compared to team athletes, both before (training, 1.0 h; *Z* = 2.606; *p* = 0.009, learning, 1.8 h; *Z* = 2.863; *p* = 0.004) and during lockdown (training, 1.3 h; *Z* = 3.577; *p* < 0.001; learning, 2.5 h; *Z* = 4.374; *p* < 0.001). There were no statistically significant differences for other variables assessed (Figure 7). Finally, when compared to athletes engaged in individual sports, athletes training and competing in team sports experienced significantly greater decline in training exposure (PD = 5.3%; *Z* = −2.349; *p* = 0.019) from PRE_LD_ and DUR_LD_ (individual sports ∆ = −4.60 ± 6.11 h. vs. team sports ∆ = −4.85 ± 6.30 h.) (Figure 3C).

**Figure 8:**
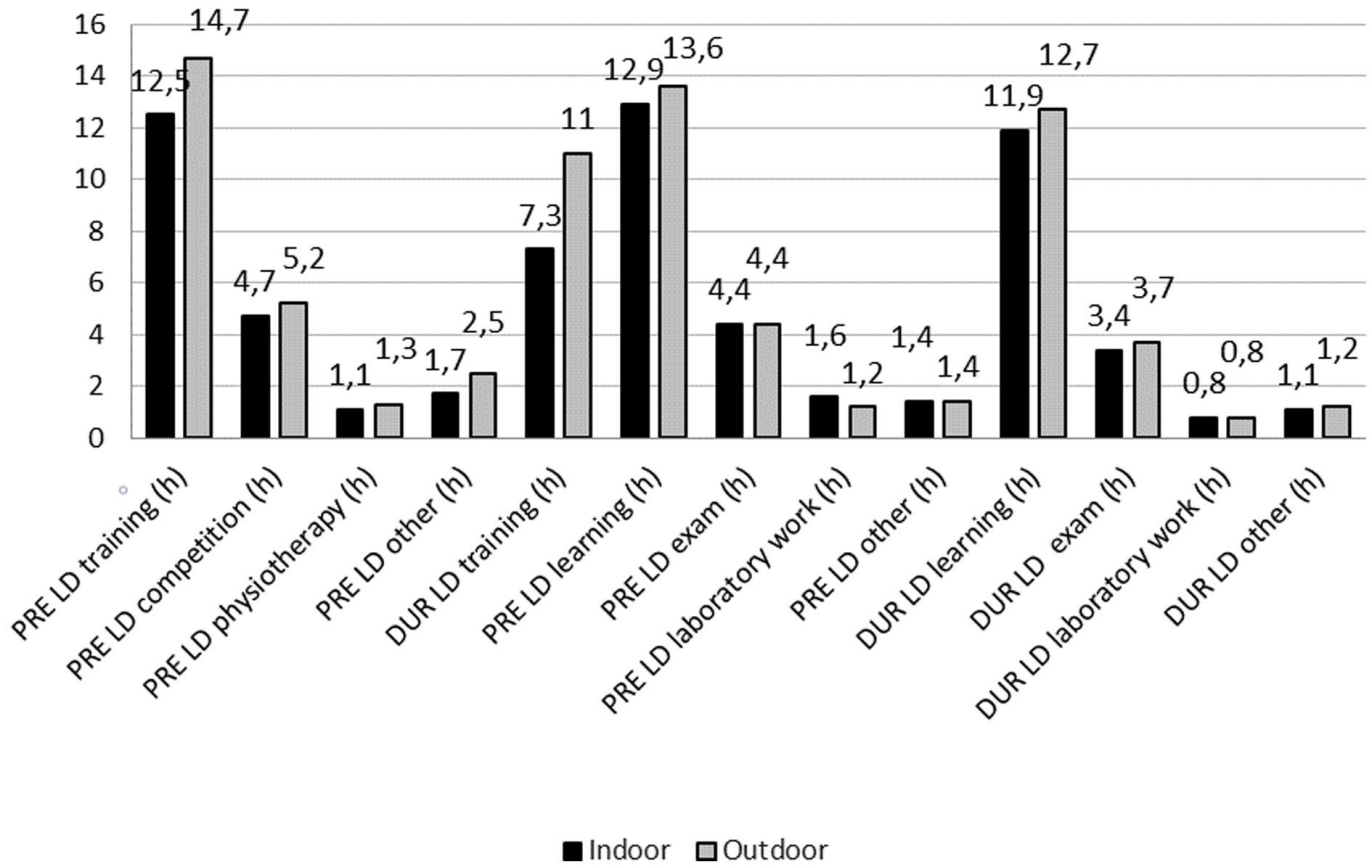
Comparisons of time spent on sport and educational activities before (PRE_LD_) and during (DUR_LD_) lockdown between athletes competing in indoor and outdoor sports.

When comparing differences between athletes engaged in indoor and outdoor sports, the results showed that athletes training and competing indoors spent less time training both before (2.2 h; *Z* = −6.382; *p* < 0.001) and during lockdown (3.7 h; *Z* = −10.847; *p* < 0.001) (Figure 8). Compared to athletes engaged in outdoor sports, athletes training and competing indoors experienced significantly greater decline in training exposure (PD = 31.7%; *Z* = −4.993; *p* < 0.001) from PRE_LD_ and DUR_LD_ (outdoor sports ∆ = −3.75 ± 5.49 h. vs. indoor sports ∆ = −5.17 ± 6.45 h.) (Figure 3D).

### Alterations in DC athletes’ training

Table 2 shows athletes’ training changes due to COVID-19 lockdown. Most of the athletes (92.9%) trained at home during the COVID-19 pandemic. Other changes included training outdoors, training at home with online support, or other changes, which were combinations of the above-mentioned alterations. Among athletes who did not train during the pandemic, nearly a third stated they lacked motivation, while others claimed they did not have adequate training conditions or listed other reasons. Most of the DC athletes (63.3%) felt they responded better to the COVID-19 pandemic than non-student athletes. Their responses indicated a smaller decline in motivation (44.8%), shifting attention from sport to study (31.3%), fewer mental problems due to uncertain sports future (15.9%) and other (8%). The DC athletes who indicated that they did not respond better during the COVID-19 pandemic (*n* = 568) listed the following reasons: failed to focus on either school or sports (28.2%), feeling bad psychologically (16.5%) and other (18.3%), while 37% trained the same as before the lockdown (Table 2).

**Table 2:** Alterations in DC athletes’ training due to COVID-19 lockdown.

		Count	%
Did you train at home during quarantine due to the COVID-19 pandemic?	Yes	1,288	92.9%
	No	99	7.1%
If yes: How was your training changed? If no: go to the next question	I trained at home	520	39.8%
	I trained outside	471	36.1%
	I trained at home with “online” support	221	16.9%
	Other	93	7.1%
If not: why you did not train:	I did not have adequate conditions for training	36	27.3%
	Lack of motivation	42	31.8%
	I felt bad physically	1	0.8%
	I felt bad psychologically	7	5.3%
	Other	46	34.8%
Do you think you responded to COVID-19 pandemic better than non-student athletes due to the combination of education and sport?	Yes	878	63.3%
	No	509	36.7%
Why has the combination of education and sports helped you cope better with the COVID-19 pandemic compared to non-student athletes?	Smaller decline in motivation	418	44.8%
	Shifting attention from sport to study	292	31.3%
	Less mental problems due to uncertain sports future	148	15.9%
	Other	75	8.0%
If no: Why the combination of education and sports has not helped you cope better with the COVID-19 pandemic compared to non-student athletes?	I failed to focus on either school or sports	160	28.2%
	I felt bad psychologically	94	16.5%
	I trained the same as before the lockdown	210	37.0%
	Other	104	18.3%

## Discussion

Dual career athletes, who are pursuing both athletic and academic careers simultaneously, faced unique challenges during the pandemic. They had to balance training and competition schedules with academic responsibilities that have been disrupted by the pandemic and also faced logistical challenges related to travel and the use of training facilities. Our research showed that the COVID-19 lockdown had a major impact on DC athletes’ lives due to several governmental measures. Compared with time spent on training and study before the lockdown, DC athletes spent less time on both during the lockdown. The biggest decline was observed in training as athletes trained 4.7 h less than before the lockdown, which is approximately a third less than usual. In relation to training and competition environment, data in our study showed that outdoor-sport athletes spent more time training before (2.2 h), but even more during the lockdown, compared to indoor-sport athletes. Those results indicate that indoor sports were definitely more influenced by governmental measures, as indoor athletes trained 3.7 h less than outdoor athletes. Both indoor and team sport athletes experienced a greater decline in training hours due to the preventive measures applied.

The preventive measures taken in Slovenia during the pandemic included limiting the number of people in indoor spaces (including gyms) and a prescribed space in m^2^ per individual, meaning that many sport clubs were unable to provide the appropriate space while conforming with the preventive measures. Therefore, they were forced to cancel, reduce, or adapt their trainings. Latter was confirmed by our results, showing that as many as 93% of athletes trained at home during the lockdown. Among athletes who claimed their training was changed, 40% of them trained at home, 36% trained outside, while others trained with online support or the combination of the mentioned options. The most common reason for not training was lack of motivation (32%), while 27% of the athletes also stated they did not have adequate conditions for training. These changes could contribute to a higher risk of injury, as the lack of sport-specific training (Verrall et al., 2005) and a low number of training sessions (Ekstrand et al., 2020) are already known to contribute to higher injury rates.

Furthermore, we found that study and training alterations were varied with the socio-demographic and sports-related characteristics of DC athletes. Males spent more time on training and sports-related activities both before and during lockdown compared to females, whereas females spent more time than males on educational activities both before and during the lockdown. This is supported by the evidence from The Organization for Economic Cooperation and Development report from 2017, which showed that completion rate in upper secondary education and both participation and completion in tertiary education is higher in female athletes than in males (Education at a Glance, 2017). Even though female sport is becoming more popular, there is still a gender gap in elite sports; for example, most female athletes around the world cannot live solely from their athletic careers (Capranica et al., 2013). Moreover, Barriopedro and colleagues found that female athletes from their study took more time than male athletes to find their first job after terminating their sports career (Barriopedro et al., 2018). Despite all that, there is also an existing gender pay gap, which is reported to be 12.7% in the European Union, in favor of men (Eurostat, 2023).

All the athletes trained less during the lockdown regardless of their competition level. Yet the governmental measures applied differently to different competition levels. Athletes competing at the prospective, international, world and Olympic levels were allowed to train the whole time, with an exception of a period when they were only allowed to conduct training sessions using a “bubble strategy.” This was shown to be a promising non-pharmaceutical intervention when coping with emerging infectious diseases (Shen et al., 2022). Sports teams intentionally established a “protective bubble” through which they restricted physical closeness with those outside of the bubble, thereby minimizing the risk of infection (Shen et al., 2022). The sub-analysis showed that individual-sport athletes spent more time training and studying both before and during the lockdown compared to team-sport athletes—a difference influenced by governmental measures. With respect to the training and competition environment, the data indicated that outdoor-sport athletes spent more time training before (2.2 h), but even more during the lockdown, compared to indoor-sport athletes. This indicates that indoor sports were more directly influenced by governmental measures about indoor containment, as indoor athletes trained 3.7 h less than outdoor athletes. However, research also shows that despite close proximity interactions in team sports, SARS-CoV-2 transmission is limited during team sport activities played outdoors (Jones et al., 2020; Dixon et al., 2021; Egger et al., 2021; Faude et al., 2022). Interestingly, research on transmission in indoor sports is inconclusive. For example, a study of 1825 water polo athletes found that transmission of the disease was minimal in indoor sports settings as well (Kreienkamp et al., 2022), but other research suggests that viral transmission in indoor sports is higher compared to outdoor sports and those sports in which preventive hygiene measures could be maintained (Pauser et al., 2021).

The pandemic had a significant impact on mental health, and athletes were no exception (Mehrsafar et al., 2021; Jia et al., 2022). DC athletes may be particularly vulnerable to psychological effects of the pandemic due to the added stressors of their dual careers (Drole et al., 2023). Understanding their responses to the pandemic can help identify risk factors for mental health issues and inform interventions to support their well-being. Most of the DC athletes think they responded better to the pandemic situation compared to non-student athletes, because they experienced less decline in motivation. It is well-known that participation in organized sport has many benefits, not only physical, but also social and psychological (Bartko and Eccles, 2003; Holt et al., 2011; Eime et al., 2013; Piepiora, 2021; Piepiora et al., 2022). Sport allows students to acquire many transferable skills, such as better self-esteem, organizational and interpersonal social skills. Athletes are known by extreme resilience, which is a characteristic that allows them to persevere motivation and bounce back faster and easier (Bartko and Eccles, 2003; Fletcher and Sarkar, 2012). They build this skill throughout their sports career, as a result of winning and losing at the competitions. Losing is an essential part of sport, as it teaches athletes to overcome disappointment, adapt to challenges and cope with unpleasant events. Playing sport can in fact help students to control their emotions and channel negative feelings in a positive way (Holt et al., 2011). All those skills can be later transferred to other areas of their life, including the times of pandemic. The DC athletes from our study felt they could also shift their focus from sport to education and felt less insecure about their future, knowing they are pursuing higher education. Overall, studying the response of DC athletes to the COVID-19 pandemic can provide valuable insights into the unique challenges they face and inform interventions to support their well-being and performance during and beyond the pandemic. DC was shown to benefit athletes even when they decide to retire from sport or get injured (Stambulova et al., 2009, 2021; Barriopedro et al., 2018; Ivarsson et al., 2018). Pursuing a DC allows athletes to diversify their interests and skills beyond their sport, which can help maintain motivation and a sense of identity outside of their athletic environment. This can be particularly important during a traumatic injury that may prevent them from participating in their sport for an extended period (Ivarsson et al., 2018). DC allows athletes to continue working towards meaningful goals despite not being able to participate in their sport. Pursuing a DC can provide athletes with socio-economic stability, which can help reduce stress and anxiety related to their injury and at the same time maintain motivation and focus on recovery and rehabilitation.

However, the lockdown measures were not only concerning during the period of pandemic, but also after the athletes transitioned to normal training and competition. Reduced training hours could have caused detraining, which is particularly concerning when athletes return to a pre-lockdown training schedule. The return to normal training could increase their training and competition load, exposing athletes to a greater risk of injury (Eirale et al., 2020; Sarto et al., 2020). Additionally, we found that the psychological state of the DC athletes was altered during the lockdown period, as some of them reported poor psychological health and that they could not focus on either sport or school. Yet it is not known how many athletes ended their sports-career during that period due to lack of motivation, financial/material support for training, or other reasons. This issue requires further research.

## Limitations

Although this study presents data from a large sample, it has a few limitations, which we have acknowledged during the implementation of the questionnaire. Firstly, the questionnaire only collected limited data, therefore it lacks details about the psychological impact and injury occurrence prior and during the lockdown period, which could provide us more valuable results. To provide even more context, it would be useful to have the post-lockdown data of the same individuals. Additionally, the pre-lockdown data relied on recall and athletes’ personal diaries, which might have impacted the results.

## Conclusion

During the COVID-19 lockdown, athletes training experienced detrimental changes in the frequency/duration domain, environment and type of training. In general, athletes trained a third less time than usual, and most athletes changed their training environment to at home or outdoors. Indoor and team sports were more affected by the governmental measures than outdoor and individual sports. DC is shown to be beneficial for athletes even in times of COVID-19 lockdown, as DC athletes reported a smaller decline in motivation by shifting attention from sport to study and they felt fewer mental problems due to the uncertainty of their sports future. Considering the health crises induced by COVID-19 outbreak, that will most likely have a long-lasting effect, our findings provide policy makers and athletes’ support staff useful information when forming and applying preventive measures that improve DC athletes’ training and education. Also, the promotion of higher education among athletes and their greater involvement in the learning processes, may serve as a potential tool for maintaining motivation and mental health among athletes in future pandemics or situations like traumatic injury or retirement from sports.

## Data availability statement

The raw data supporting the conclusions of this article will be made available by the authors, without undue reservation.

## Ethics statement

The studies involving human participants were reviewed and approved by Ethics Committee of the Faculty of Sport (University of Ljubljana). The patients/participants provided their written informed consent to participate in this study.

## Author contributions

KD drafted the manuscript and contributed to the conceptualization. AP contributed to conceptualization, design, and data analysis. JC critically revised the manuscript, while MD contributed to the idea and critically revised the manuscript. All authors contributed to the article and approved the submitted version.

## Funding

The research took place within the Kinesiology of Monostructural, Polystructural and Conventional Sports research program, code: P5-0147, financed by the Public Research Agency of the Republic of Slovenia. The funding agency has no impact on data collection, analysis or interpretation of the study results.

## Conflict of interest

The authors declare that the research was conducted in the absence of any commercial or financial relationships that could be construed as a potential conflict of interest.

## Publisher’s note

All claims expressed in this article are solely those of the authors and do not necessarily represent those of their affiliated organizations, or those of the publisher, the editors and the reviewers. Any product that may be evaluated in this article, or claim that may be made by its manufacturer, is not guaranteed or endorsed by the publisher.

## Footnotes

^1^ https://www.1ka.si/
